# Neutrophilic leukocytosis and erythema nodosum leprosum in leprosy: insights from a retrospective observational study

**DOI:** 10.3389/fimmu.2024.1368460

**Published:** 2024-07-12

**Authors:** Marcella Feitosa da Silva Barboza, Mariana de Andrea Hacker, Anna Maria Sales, Débora Fontoura Rodrigues, Daniel Pedrosa Marques, Danillo José Ciryllo Silva Noya, Thabatta Leal Silveira Andrezo Rosa, Isabel de Fátima Alvim Braga, Helen Ferreira, Thais Porto Amadeu, Monique Gurgel de Oliveira, Alice de Miranda Machado, Ximena Illarramendi, Veronica Schmitz

**Affiliations:** ^1^ Laboratório de Hanseníase, Instituto Oswaldo Cruz (IOC), Fundação Oswaldo Cruz (Fiocruz), Rio de Janeiro, Brazil; ^2^ Instituto Nacional de Infectologia Evandro Chagas (INI), Fundação Oswaldo Cruz (Fiocruz), Rio de Janeiro, Brazil; ^3^ Programa de Pós-graduação em Medicina Tropical (PGMT) Instituto Oswaldo Cruz (IOC), Fundação Oswaldo Cruz (Fiocruz), Rio de Janeiro, Brazil; ^4^ Laboratório de Microbiologia Celular, Instituto Oswaldo Cruz, Fundação Oswaldo Cruz, Rio de Janeiro, Brazil; ^5^ Coordenação Geral de Gestão de Pessoas (Cogepe), Fundação Oswaldo Cruz (Fiocruz), Rio de Janeiro, Brazil; ^6^ Laboratório de Imunopatologia, Faculdade de Ciências Médicas, Universidade do Estado do Rio de Janeiro (UERJ), Rio de Janeiro, Brazil; ^7^ Centro para o Desenvolvimento Tecnológico em Saúde (CDTS), Fundação Oswaldo Cruz (Fiocruz), Rio de Janeiro, Brazil

**Keywords:** neutrophilic leukocytosis, erythema nodosum leprosum (ENL), leprosy reactions, NLR, neutrophil

## Abstract

**Background:**

Leprosy reactions represent immunologically mediated episodes of acute inflammation that, if not diagnosed and treated promptly, can cause irreversible impairment of nerve function and permanent disabilities. A frequent type of reaction experienced by patients with lepromatous leprosy (LL) and borderline lepromatous leprosy (BL) is erythema nodosum leprosum (ENL), an inflammatory complication that may become chronic or recur in multiple episodes. Although ENL is commonly described as a neutrophil-mediated immune disease, the role of neutrophils is not fully understood. In this study, we assess neutrophilic leukocytosis in a retrospective cohort of patients affected by BL or LL leprosy.

**Materials and methods:**

A retrospective observational study was performed using data from 146 patients with BL and LL leprosy diagnosed and treated at the Souza Araújo Outpatient Clinic, Fiocruz, Rio de Janeiro, Brazil. Clinical, demographic, and hematological data were extracted from medical records. Skin biopsy samples obtained from patients for ENL diagnosis were used for histopathological evaluations.

**Results:**

Most patients were male (75%) and had a reactional episode (85%), of which 65% were ENL. Multiple episodes were common, 55% of the 80 patients with ENL presented more than 2 episodes (average of 2.6 episodes). In treatment-naive BL/LL patients, the median blood neutrophil counts of patients who developed ENL at some points of their disease course were higher than those who did not experience any reaction (median= 4,567 cells/mm3 vs 3,731 cells/mm3 respectively, p=0.0286). A correlation between the increase in median neutrophil counts and ENL severity was confirmed (6,066 cells/mm3 for mild ENL vs 10,243 cells/mm3 for moderate/severe ENL, p=0.0009). A longitudinal assessment was also performed in 34 patients, confirming the neutrophilic leukocytosis (BL/LL: 4896 cells/mm3 vs ENL: 8408 cells/mm3, p<0.0001). Moreover, increased NLR was associated with a greater neutrophilic infiltration in ENL lesions.

**Conclusion:**

We demonstrate that ENL episodes in patients affected by leprosy are associated with elevated blood leukocyte and neutrophil counts and an increased NLR. These findings highlight the significant involvement of neutrophils in the ENL immunological/inflammatory process.

## 1 Introduction

Leprosy, also known as Hansen’s disease, is a neglected tropical disease that still occurs in over 180 countries, with more than 170,000 new cases reported every year (1). Brazil reported 19,635 new cases diagnosed in 2022 and has the highest number of cases in the region of the Americas (2). The disease has been attributed to both *Mycobacterium leprae* and *M. lepromatosis* (3), with most cases caused by *M. leprae*, an obligate intracellular acid-fast bacillus (AFB), which multiplies slowly resulting in a chronic disease course (4). The location of leprosy lesions on the patient’s body (skin, nasal mucosa, and peripheral nerves) is associated with the bacillus tropism for Schwann cells and skin macrophages (5).

Individuals infected with *M. leprae* present a wide spectrum of clinical and histopathological manifestations that vary according to the intensity of the individual’s immune response to the infection. These manifestations have already been subjected to different classifications; however, a consensus exists that the primary classification should be made based on the clinical appearance of the skin lesions and neurological manifestations (4).

Ridley and Jopling’s (1966) classification uses clinical, bacteriological, immunological, and histopathological criteria, with emphasis on the last criterion. The clinical forms of the disease fall within a clinical spectrum that varies from tuberculoid leprosy (TT) to lepromatous leprosy (LL). TT characterized by few skin lesions, with absent bacilloscopic index (BI), where patients have a strong immune response mediated by Th1 cells, and LL is characterized by multiple disseminated skin lesions, a high BI, with Th2 type immunity and vigorous production of antibodies. Three intermediate forms, known as borderline tuberculoid (BT), borderline-borderline (BB), and borderline lepromatous (BL), in which patients have some cell-mediated immune response, a variable number of skin lesions and unstable immunity, combining features of both poles (6).

Differences in the degree of cellular immune response to M. leprae are responsible for the different types of granulomatous reaction; epithelioid cells are usually seen in the skin lesions of TT and BT patients, whereas foamy macrophages are found in those of BL and LL patients (7).

Leprosy treatment in Brazil follows the Ministry of Health (MoH) recommendations and is based on the combination of three medications, known as multidrug therapy (MDT): rifampicin, dapsone, and clofazimine. Patients classified with BL and LL leprosy take 12 directly observed monthly doses of 600 mg of rifampicin and daily doses of 100 mg of dapsone and 300 mg of clofazimine for a period of 12-18 months (8).

Even though the MDT available is effective in treating leprosy, one of the main difficulties in the clinical management of patients is that around 30–40% can develop acute episodes of inflammatory response, called leprosy reactions, which can occur before diagnosis, during treatment, and even years after treatment release (9). Leprosy reactions represent immunologically mediated episodes of acute inflammation that, if not diagnosed and treated promptly, can accelerate nerve damage and permanent disability. There are two main types of leprosy reaction: type 1 reaction or reversal reaction, (RR), which is associated with delayed-type hypersensitivity reactions, resulting from increased cell-mediated immunity to M. leprae antigens; and type 2 reaction, represented mainly by erythema nodosum leprosum (ENL), which is described as a neutrophilic immune complex-mediated disease (10, 11).

ENL is a multisystem complication that presents painful, erythematous skin nodules frequently together with fever, malaise, and neutrophilic leukocytosis. It occurs in around 50% of LL patients and 5–10% of BL patients (12). Treatment involves high doses of corticosteroids or thalidomide taken for months or years, depending on drug availability and patient condition. Few patients experience only one acute episode of ENL, while the vast majority have recurrent episodes and chronic illness. Furthermore, patients often experience multiple adverse events owing to the prolonged use of corticosteroids, leading to a severe socioeconomic impact, an increase in morbidity and mortality, and quality of life reduction (13, 14).

The cause of ENL is complex and the cellular and molecular mechanisms that trigger and sustain ENL are not fully understood. Neutrophils are considered one of the main markers of ENL, as intense perivascular infiltration of neutrophils is often observed throughout the deep layers of the dermis and subcutaneous tissue of ENL lesion biopsy histopathology (15). However, not all clinically confirmed cases of ENL present neutrophilic infiltration in the lesions (16), and the timing of the skin biopsies appears crucial in detecting neutrophil infiltration (17).

Circulating neutrophils and monocytes are loaded with bacilli in the blood of BL and LL patients, and their clearance only effectively occurs after 2–3 months of MDT (18) Patients with ENL present high neutrophilia and changes in the neutrophil-to-lymphocyte ratio (NLR) (19). However, although neutrophilic leukocytosis is always cited as a characteristic component of ENL, this is seldom reported in the literature.

Currently, no laboratory test has been found to predict the emergence of leprosy reactions in newly diagnosed patients. This study, therefore, involves a retrospective survey of laboratory test results of patients diagnosed with BL or LL leprosy to identify a potential predictive indicator for the emergence of ENL.

## 2 Materials and methods

### 2.1 Study design and patients groups

**Figure 1:**
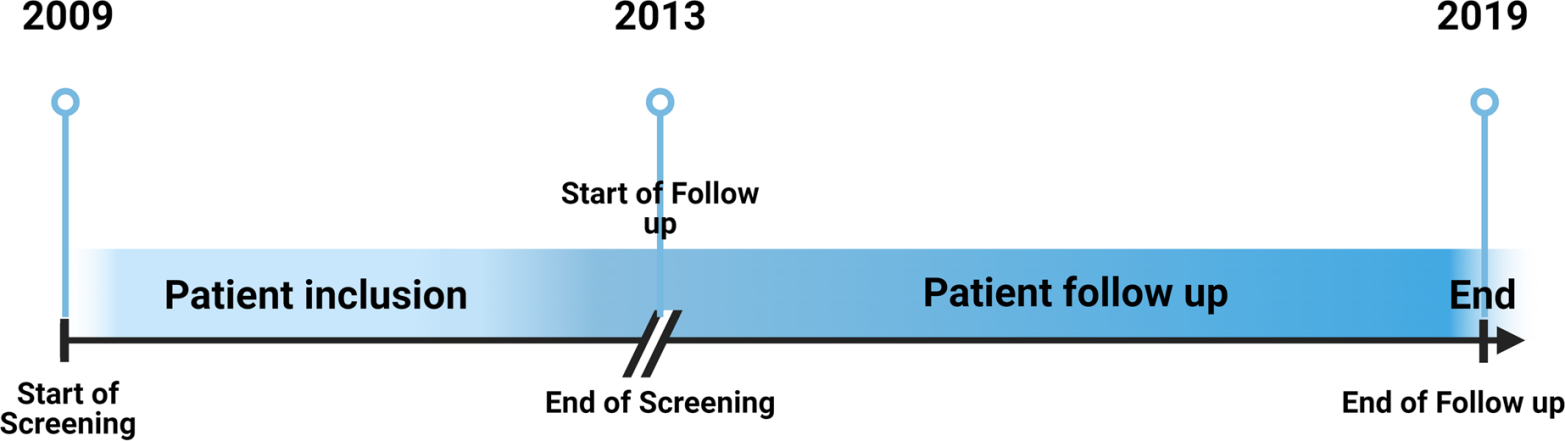
Study data collection timeline. Patients diagnosed with BL or LL leprosy between January 2009 and December 2013 (Inclusion period) had their clinical, demographic, and hematological data collected. Information regarding ENL episodes (clinical and hematological data) were continued collected from these patients until December 2019 (Follow-up period).

This observational retrospective study was conducted at the Souza Araújo Outpatient Clinic (ASA), a reference center for leprosy diagnosis and treatment [Leprosy Laboratory, Oswaldo Cruz Foundation (Fiocruz), Rio de Janeiro, Brazil]. Clinical, demographic, and hematological data of patients 18 years old or above, classified as BL or LL, and diagnosed between January 2009 and December 2013 (Inclusion period). Information regarding ENL episodes (clinical and hematological data) were collected from these patients until December 2019 (Follow-up period). Those data was collected from the electronic data system (ASA system), where all patient data are recorded (**Figure 1**).

**Figure 2:**
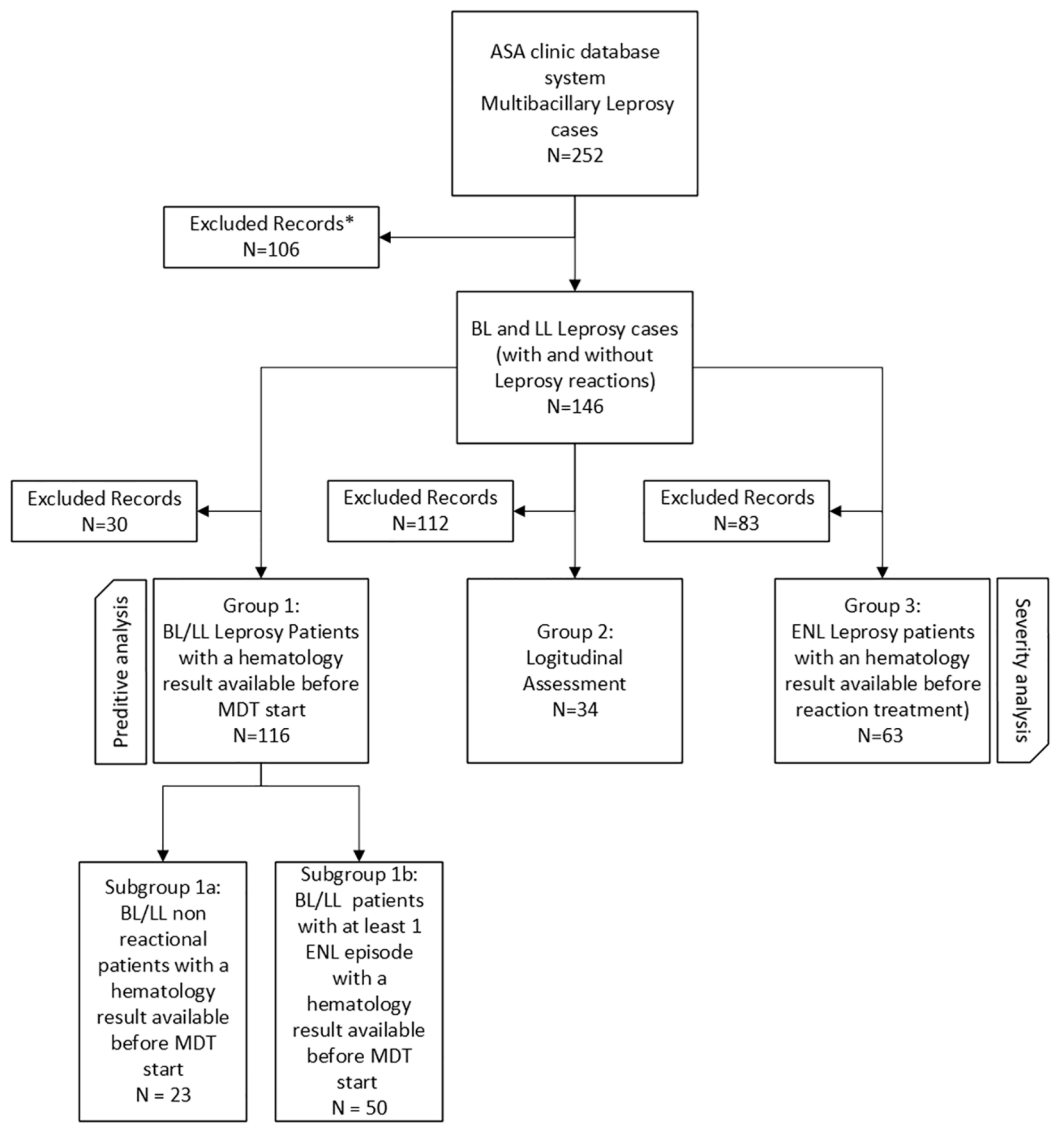
Study design flowchart. The data of 252 individuals affected by lepromatous leprosy (LL) and borderline lepromatous leprosy (BL) were collected from the clinic database. After exclusions 146 patients were selected for the study. Three groups were designed - two cross-sectional and one longitudinal. *Excluded records were considered: <18 years (n=8), BB cases (n=4), HIV (n=1), pregnancy (n=1), absence of medical records (n=5), treatment abandonment (n=10), transfer to another unit (n=10), death (n=3) and absence of laboratory results (n=64). Group 1 (n=116) comprised patients (with and without a history of leprosy reactions but not an active ENL at the time of admission) with a hematology result available before the start of MDT. To perform predictive analysis, patients of Group 1 were further divided into two subgroups: a group of 23 patients who did not experience any reactional episode during the study follow-up period (Subgroup 1a) and a group of 50 patients who experienced at least one ENL episode during the study follow-up period (Subgroup 1b). Group 2 (n=34) was used for a longitudinal assessment of patients with hematology results before the start of MDT and at the onset of an ENL reaction, with 34 patients who experienced a total of 50 ENL episodes. Group 3 (n=63) comprised patients who experienced at least one ENL reaction during the follow-up period and a hematology result available at the onset of the ENL reaction. To perform severity analysis, the ENL reaction severity was determined according to an ENL Severity Scale applied based on the EESS.

The whole contents of the patient charts, from the admission to the clinic until the last visit were reviewed, as well as the available skin biopsy fragments from ENL lesions. The study was approved by the Fiocruz/IOC Human research ethics Review Board (CAAE 40459620.6.0000.5248). An informed consent waiver was requested and approved, and eligibility for inclusion was patients aged 18 years and above. The study design is represented in the flowchart in **Figure 2**. Three groups were formed according to the hematological laboratory results available: Group 1, patients with results available before MDT; Group 2, a subset of group 1 formed by patients that had hematologic results also at the beginning of each ENL episode; Group 3, patients with ENL reactions, with or without neuritis, and laboratory results during reaction.

At ASA, leprosy diagnosis is based on clinical signs and symptoms, AFB in skin smears and histopathology of skin or nerve biopsies are analyzed at the Leprosy Laboratory. Patients are classified using the Ridley and Jopling criteria. The Ridley-Jopling classifications are clinically determined by examining the morphology of skin lesions and nerve involvement patterns, along with the BI, and supported by histologic examination of a skin biopsy specimen (6). BL leprosy patients manifest multiple and symmetrical lesions that initially appear as hypopigmented macules with indistinct borders, which gradually merge into the normal skin. As the disease progresses the macules become infiltrated and form plaques and nodules. LL leprosy patients present an uncontrolled multiplication of bacilli caused by the severe weakening of cell-mediated immunity. AFBs are highly positive. Lesions of LL are numerous and exhibit bilateral symmetry over the face, extremities, and trunk (20).

Before MDT, blood hematology and biochemistry analyses at the onset of ENL are also performed for most patients. These laboratory tests are performed as a routine service by the clinical analysis laboratory of the Evandro Chagas National Infectology Institute (INI-Fiocruz).

The case definition of ENL was a diagnosed leprosy patient with an acute appearance of crops of tender cutaneous or subcutaneous nodules, accompanied or not by fever, malaise, or other systemic involvement. RR was defined by the exacerbation of pre-existing skin lesions and the appearance of new plaques with an inflammatory appearance. Neuritis was defined clinically by the presence of nerve thickening and/or tenderness, accompanied by acute loss of sensory and/or motor neural function, and by the presence of demyelinating lesion under a nerve conduction study.

Patients were treated with MDT, as recommended by the Brazilian MoH. Those patients with known allergies or drug intolerance received substitutive MDT, that is composed either ofloxacin, minocycline and/or clarithromycin. ENL, RR, and neuritis were treated in compliance with Brazilian MoH guidelines (8).

### 2.2 Severity of ENL (collected from the medical charts)

ENL severity was determined after the review of the reactions registered in the medical charts. Each reaction was reviewed and scored in accordance with the following criteria: Presence or absence of fever, number of skin lesions, inflammation of skin lesions, extent of skin lesions, presence or absence of peripheral oedema, inflammation of joints and/or digits due to ENL, lymphadenopathy due to ENL, nerve tenderness due to ENL. Mild ENL was characterized by less than 10 tender skin lesions, often restricted to a few regions, no fever, and undefined aches and pains. Moderate ENL was characterized by 10–20 nodules, which are painful on palpation, in association with a moderate fever (<38.4°C) and discrete systemic symptomatology, possibly affecting local and/or regional lymph node chains. Severe ENL is characterized by more than 20 painful nodules, often with vesicular or ulcerated lesions involving a large area of tegument (more than 5 regions), accompanied by expressive systemic symptomatology, such as a high fever (>38.5°C), arthralgia, fatigue, and involvement of lymph node chains. This retrospective classification was based in the ENLIST ENL Severity Scale (EESS) developed and validated by Walker et al. (12, 21). The EESS was not applied in this study because the data was obtained before the ENList scale publication.

ENL type categorization was also performed retrospectively, and following Walker et al. (2014) definitions: acute, recurrent, and chronic. Acute ENL was defined as a single episode lasting less than 24 weeks. Recurrent ENL was defined as a second or subsequent episode of ENL occurring 28 days or more after stopping treatment for ENL. Chronic ENL was defined as occurring for 24 weeks or more during which a patient has required ENL treatment either continuously or where any treatment free period had been 27 days or less (22).

### 2.3 NLR categorization

The NLR values obtained from ENL patients were categorized according to the NLR meter proposed by Zahorec in 2021 (23). The four categories were defined as follows: Category 1 (NLR >1.5-3.5), Category 2 (NLR > 3.5-7.5), Category 3 (NLR >7.5–14), and Category 4 (NLR >14).

### 2.4 Classification of neutrophilic infiltrate

Skin biopsy specimens containing both the epidermis and dermis of an active ENL lesion were reassessed to classify neutrophilic infiltrates. Histopathological analysis was conducted on samples fixed in 10% formalin for at least 24 hours. The laboratory’s standard procedure was followed by embedding the samples in paraffin. Serial sections in the sagittal plane at a thickness of 5 μm were performed using a Leica microtome. Hematoxylin and Eosin (HE)-stained sections were analyzed under a Nikon Eclipse microscope, and images were obtained with Opticam Microscopia OPTHD software.

A semi-quantitative blind analysis was performed in five high-magnification fields (HMF) (40x objective) by two experienced pathologists in parallel. They analyzed the dermal skin area with inflammatory infiltrates and recorded the infiltrate with prominent polymorphonuclear cells (PMNs). The agreement rate between pathologists was 96%. During the analysis of the inflammatory infiltrate, the focus was on examining the deep dermis and subcutaneous tissue, as they are the primary areas where the neutrophil count is found in ENL.

The degree of PMN infiltration was classified into the following categories: i) Mild infiltration: less than 10% of the sample with inflammation, with less than 10 PMN per HMF; ii) Moderate infiltration: between 10% and 50% of the sample with inflammation, regardless of the number of neutrophils or more than 50% of the sample with inflammation and between 10 and 20 PMN per HMF; and iii) Intense infiltration: more than 50% of the dermis with inflammation, with more than 20 PMN per HMF.

### 2.5 Immunohistochemical staining

ENL paraffin skin tissue sections (4 μm) were analyzed by the immunoperoxidase method. Briefly, tissues were dewaxed in xylene and hydrated in ethanol. Antigen retrieval was done using Target Retrieval Solution (DAKO, S1699) followed by blocking with 10% normal goat serum. Sections were incubated with monoclonal anti−Human PTX3/Pentraxin 3 antibody (clone MNB1, Cat. N. LS−C140141 human monoclonal antibody against to pentraxin-3 (LS Bio, 1:50) and appropriate isotype control, overnight at 4°C, with subsequent incubation by HiDef HRP detection Kit (Cell Marque, 954D-31-RUO). Detection was revealed using diaminobenzidine chromogen (DAKO, K3465). Sections were counterstained with Mayer’s hematoxylin and mounted. Images were obtained via Nikon Eclipse microscope with Opticam Microscopia OPTHD software.

### 2.6 Study variables

The following information were assessed in this study:

Demographic variables: sex and age.

Clinical variables: BL or LL at diagnosis, as according to Ridley-Jopling classification (Ridley and Jopling, 1966), and MDT details, such as treatment period (start and end date) and regimen.

Leprosy reactions: Presence or absence of leprosy reaction, reaction treatment details, and the ENL Severity classification applied as described above.

Hematological variables: BI, red blood cell (RBC) count, hemoglobin level, hematocrit level, mean corpuscular volume (MCV), mean corpuscular hemoglobin (MCH), mean corpuscular hemoglobin concentration (MCHC), white blood cell (WBC) count, and basophils, eosinophils, neutrophils, lymphocytes, monocytes, and platelets counts. Additionally, the NLR was calculated by dividing neutrophil count/lymphocyte count for all patients with available results.

Data collected from the outpatient clinic electronic data system, ASA system, and from patient charts were managed using Research Electronic Data Capture (REDCap 13.7.22 © 2023 Vanderbilt University), a secure web platform resource hosted at IOC.

### 2.7 Statistical analysis

Statistical analyses were performed in GraphPad Prism version 9 (GraphPad Software, San Diego, CA, USA). Descriptive analysis was initially performed for all study variables, and values are reported as mean, median, and standard deviation. The Shapiro–Wilk test was used to verify normality for the distribution of the hematological results. The Mann–Whitney test was used to compare continuous variables between groups. The Wilcoxon matched-pairs signed-rank test was used to compare paired samples. The adopted statistical significance level was p <0.05. The optimal cutoff values for the total WBC count, neutrophil count, and NLR were determined from receiver operating characteristic (ROC) curve analysis. According to this analysis, a test that gives an area under the curve (AUC) above 0.7 is considered satisfactory (24).

## 3 Results

### 3.1 Overview of the demographic, clinical, and hematological profiles of patients with BL/LL leprosy

Over the period studied, a total of 252 patients diagnosed with BL or LL leprosy were registered in the ASA system; 106 were excluded from the analysis owing to age, HIV co-infection, pregnancy, MDT non-compliance or absence of laboratory results (**Figure 2**). In total, 146 patients with leprosy were selected for the study. From this total, three groups were designed - two cross-sectional groups and one longitudinal cohort. Group 1 (n=116) comprised patients (with and without a history of leprosy reactions but not an active ENL at the time of diagnosis) with a hematology result before the start of MDT. Group 2 (n=34) involved patients with hematology results before the start of MDT and at the onset of an ENL reaction for longitudinal assessment. Group 3 (n=63) comprised patients who experienced at least one ENL reaction during the follow-up period with a hematology result available at the onset of the ENL reaction.

In Group 1 (BL/LL group; 116 patients), 75% were male and 25% were female, with more than 80% of patients aged 18–60 years. Sixty-four patients (55%) had LL and 52 (45%) had BL. A BI equal to or greater than 3 was observed in 73.3% of patients. Standard MDT was used in 92 patients (79%) and leprosy reactions were observed in 80% of the patients in this group, with more than 50% of these reactions being ENL (**Table 1**).

**Table 1:** Demographic and clinical characteristics of the 146 patients treated at the Souza Araújo Outpatient Clinic between January 2009 and December 2019 included in the study – Group 1 (n=116) and Group 2 (n=34).

Characteristics	Group 1	Group 2
	Patients with at least one hematology laboratory result available before MDT	Longitudinal Assessment
n (sample size)	116	34
Sex - n° (%)		
Male	87 (75)	29 (85)
Female	29 (25)	5 (15)
Age – n° (%)		
18–30	25 (22)	12 (35)
31–60	68 (59)	17 (50)
>60	23 (20)	5 (15)
Leprosy information		
Clinical form – n° (%)		
Borderline lepromatous leprosy (BL)	52 (45)	9 (26)
Lepromatous leprosy (LL)	64 (55)	25 (74)
Bacilloscopic index – n° (%)		
>3.0	85 (73)	32 (94)
≤3.0	31 (27)	2 (6)
Multidrug therapy – n° (%)		
Standard	92 (79)	25 (74)
Substitutive	24 (21)	9 (26)
Presence of leprosy reaction during the disease course – n° (%)		
Yes	93 (80)	–
No	23 (20)	–
Type of leprosy reaction – n° (%)		
Erythema nodosum leprosum (ENL)	50 (53.8)	–
Reversal reaction (RR)	40 (43)	–
Neuritis	3 (3.2)	–

In Group 2 (longitudinal assessment; 34 patients), 85% were male, and 15% were female, with 85% of patients aged 18-60 years. 74% had lepromatous leprosy (LL) and 94% of the patients had a BI value greater than 3. Standard MDT was used in 74% patients in this group (**Table 1**).

In group 3 (ENL group; 63 patients), which comprised patients who experienced at least one ENL reaction, 50 (79%) were male and 13 (21%) were female. Fifty-two patients (83%) were classified as LL and 11 (17%) as BL. A BI equal to or greater than 3 was observed in 59 (94%) patients (**Table 2**). A total of 169 reactions were observed in this group, most of which were classified as chronic (71%), while 78% were moderate/severe. In 72% of patients, the ENL reaction occurred during or after MDT (**Table 3**).

**Table 2:** Demographic and clinical characteristics of the 63 patients that developed ENL at some point of the disease course after leprosy diagnosis and had at least one hematology test available before, during, and/or after MDT – Group 3 (n=63).

Demographic information (Group 3 – n=63)		
Sex	n (sample size)	%
Male	50	79
Female	13	21
Age		
18–30	25	40
31–60	30	48
>60	8	12
Leprosy information		
Clinical form		
Borderline lepromatous leprosy	11	17
Lepromatous leprosy	52	83
Bacilloscopic index		
>3.0	59	94
≤3.0	4	6
Multidrug therapy		
Standard	48	76
Substitutive	15	24

**Table 3:** ENL information of the 63 patients who developed ENL at some point of the disease course after leprosy diagnosis and had at least one hematology test available before, during, and/or after MDT— Group 3 (n=63).

ENL information (Group 3 – n=63)		
Number of episodes	n	%
1 episode	26	41
2 episodes	13	21
3 episodes	13	21
>3 episodes	11	17
Duration		
Acute	17	27
Recurrent	1	2
Chronic	45	71
ENLIST Severity Assessment		
Mild	14	22
Moderate	31	49
Severe	18	29
ENL reaction occurrence		
Before MDT	18	28
During MDT	20	32
After MDT	25	40

### 3.2 Evaluating the predictive value of neutrophilic leukocytosis in ENL development

To evaluate the NLR predictive value, a comparison of results in blood counts before the start of MDT was performed. To proceed with this objective, patients of group 1 (BL/LL group) were divided into two subgroups: a group of 23 patients who did not experience any reactional episode during the disease course [subgroup 1a, named BL/LL non reactional (NR) group] and a group of 50 patients who experienced at least one ENL episode during the course of the disease [subgroup 1b, named BL/LL ENL group] and a comparison between the hematological parameters, including the NLR, for patients in the two subgroups was conducted to identify any change that could potentially predict the occurrence of ENL.

**Figure 3:**
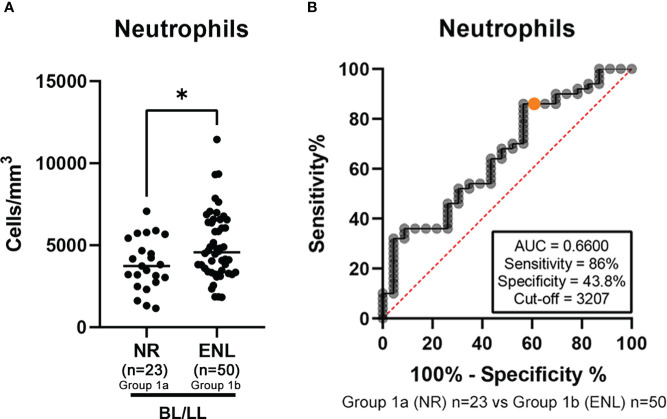
Subgroup 1a (n=23) vs Subgroup 1b (n=50) - Comparison of results in blood counts before the start of MDT and Receiver operating characteristic (ROC) curves for total neutrophil. **(A)** Total neutrophil and **(B)** ROC curves comparing total neutrophil counts in the BL/LL nonreactional [NR] subgroup 1a vs BL/LL ENL subgroup 1b. Statistical analysis was performed using a Mann–Whitney test (*p<0.05).

Mean and medians were similar between the groups for almost all parameters, except for the eosinophil and neutrophil counts (**Table 4**). Eosinophil levels from the BL/LL NR subgroup 1a were higher than those of the BL/LL ENL subgroup 1b (173.70 cells/mm^3^ vs 94.45 cells/mm^3^ respectively, p=0.0416), in contrast a higher neutrophil count was observed in the BL/LL ENL subgroup 1b compared to that of the BL/LL NR subgroup 1a (4567 cells/mm^3^ vs 3731 cells/mm^3^ respectively, p=0.0286). The statistically significant differences found between the groups for neutrophil counts (**Figure 3A**), suggested that these parameters could perhaps be involved in this prediction.

**Table 4:** Comparison of blood counts before the start of MDT - Hematological parameters for the 23 nonreactional (NR) Subgroup 1a and 50 ENL Subgroup 1b.

Parameter	BL/LL NR subgroup 1a	BL/LL ENL subgroup 1b	p value
RBC count (cells/mm³)a	4602609 ± 472683	4699796 ± 473812	0.3454
Hemoglobin (g/dL)b	13.70 (9.50–17.10)	13.70 (3.60–16.00)	0.9694
Hematocrit (%)b	40.80 (30.80–45.20)	40.65 (24.50–47.00)	0.8527
MCV (µm³)b	86.20 (72.10–102.00)	85.50 (60.80–97.90)	0.4920
MCH (pg)b	29.50 (23.10–33.50)	28.80 (16.10–33.20)	0.4693
MCHC (g/dL)b	33.30 (30.80–35.90)	33.75 (26.50–36.10)	0.5076
WBC count (cells/mm³)b	6310 (2510–9780)	6805 (4120–16340)	0.1982
Basophils (cells/mm³)b	0.000 (0.000–235.0)	0.000 (0.000–48.70)	0.6699
Eosinophils (cells/mm³)b	173.70 (0.000–1175)	94.45 (0.000–1862)	0.0416
Neutrophils (cells/mm³)b	3731 (1155–7063)	4567 (1827–11438)	0.0286
Lymphocytes (cells/mm³)b	1615 (658.0–3272)	1723 (834.0–5683)	0.3677
Monocytes (cells/mm³)b	486.9 (90.50–915.20)	531.9 (84.40–2451.00)	0.6480
Platelets (cells/mm³)b	266000 (167000–436000)	248000 (135000–1032000)	0.6464
NLRb	2.140 (0.730– 6.580)	2.870 (0.850–5.570)	0.1269

ENL, erythema nodosum leprosum; RBC, red blood cell; MCV, mean corpuscular volume; MCH, mean corpuscular hemoglobin; MCHC, mean corpuscular hemoglobin concentration; WBC, white blood cells; NLR, neutrophil-to-lymphocyte ratio; a, Parametric distribution, mean (± standard deviation); b, Non-parametric distribution, median (minimum–maximum). Non-paramertic variables were compared by Mann-Whitnney test. Parametric variables were compared by t-student test.

The accuracy of neutrophil counts for predicting the development of ENL during follow-up in patients with LL or BL was analyzed by ROC curve. AUC for neutrophil counts was 0.6600 for the prediction of ENL occurrence (**Figure 3B**), concluding that the neutrophil counts could not act as a prediction parameter for ENL occurrence. Sensitivity was 86% (95% Confidence Interval (CI): 73.81–93.05%) and specificity 43.48% (95% CI: 725.63–63.19%).

### 3.3 Neutrophilic leukocytosis as a characteristic component of ENL

Of the 146 patients initially selected, considering those with at least one hematological result before starting MDT and one result before the start of ENL treatment, 34 patients were identified and formed group 2. Those 34 patients experienced a total of 50 ENL reactions with hematological results available for analysis, which were used in the comparison (**Table 5**).

**Table 5:** Group 2 (n=34) - Longitudinal assessment of hematological parameters of BL/LL at diagnosis and at ENL diagnosis.

Parameter	BL/LL		ENL		p value
	Mean (± SD)	Median	Mean (± SD)	Median	
RBC count (cells/mm³)	4634082 ± 478931	4700000	4189898 ± 818052	4340000	0.0005
Hemoglobin (g/dL)	12.70 ± 2.608	13.60	12.19 ± 1.983	12.35	0.0743
Hematocrit (%)	39.26 ± 4.511	40.10	36.81 ± 5.134	37.65	0.0064
MCV (µm³)	84.79 ± 5.822	86.00	86.47 ± 6.936	87.90	0.0077
MCH (pg)	28.31 ± 2.580	28.70	29.69 ± 8.199	29.00	0.1558
MCHC (g/dL)	33.30 ± 1.830	33.50	33.04 ± 1.812	33.00	0.2349
WBC count (cells/mm³)	7910 ± 2860	7680	13183 ± 7708	11290	<0.0001
Basophils (cells/mm³)	3.811 ± 12.67	0.000	0.000 ± 0.000	0.000	0.1250
Eosinophils (cells/mm³)	127.1 ± 193.1	86.50	140.0 ± 255.4	0.000	0.7461
Neutrophils (cells/mm³)	5164 ± 2172	4896	10092 ± 6902	8408	<0.0001
Lymphocytes (cells/mm³)	2030 ± 1099	1659	1946 ± 1028	1766	0.3975
Monocytes (cells/mm³)	574.0 ± 345.6	531.0	724.9 ± 441.4	630.4	0.0202
Platelets (cells/mm³)	295082 ± 140216	267000	375939 ± 392216	309000	0.0722
NLR	2.839 ± 1.354	2.570	6.564 ± 5.631	3.900	<0.0001

ENL, erythema nodosum leprosum; RBC, red blood cell; a Parametric distribution, mean (± standard deviation); b Non-parametric distribution; median (minimum–maximum) MCV, mean corpuscular volume; MCH, mean corpuscular hemoglobin; MCHC, mean corpuscular hemoglobin concentration; WBC, white blood cell; NLR, neutrophil-to-lymphocyte ratio; SD, standard deviation; variables were compared by Wilcoxon test.

**Figure 4:**
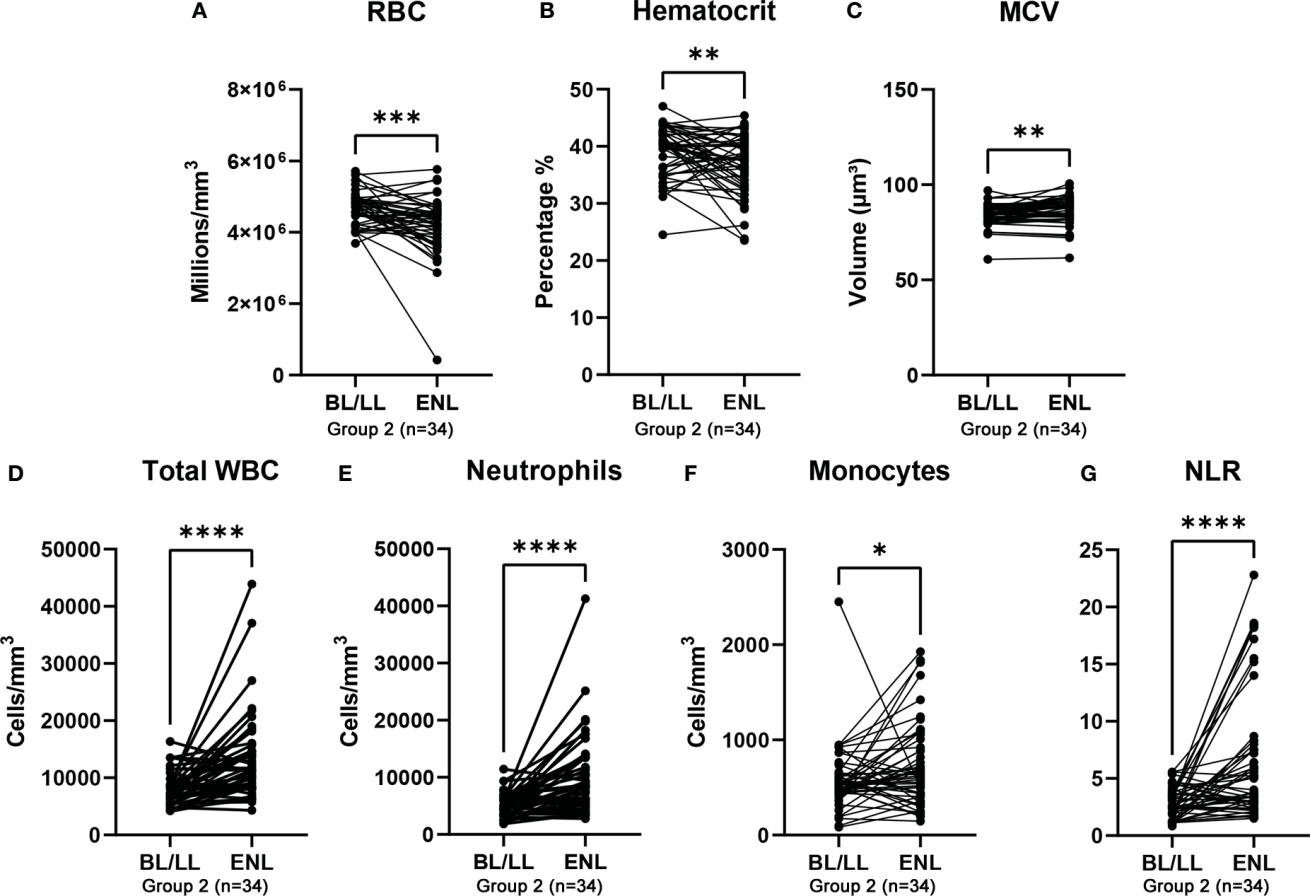
Group 2 (n=34) - Blood profile in BL/LL patients at leprosy diagnosis and at the onset of ENL. **(A)** Red blood cell (RBC) count, **(B)** hematocrit level, **(C)** mean corpuscular volume (MCV), **(D)** total white blood cell (WBC) count, **(E)** neutrophil count, **(F)** monocyte count, and **(G)** neutrophil-to-lymphocyte ratio (NLR). Symbols and lines represent individual patients. Statistical analysis was performed using a Wilcoxon matched-pairs signed-rank test (*p<0.05; **p<0.01; ***p<0.001; ****p<0.0001).

The hematological profile, in this comparison, showed statistically significant differences between the RBC counts, hematocrit levels, MCV, WBC, neutrophil, and monocyte counts, and the NLRs (**Table 5** and **Figure 4**).

**Figure 5:**
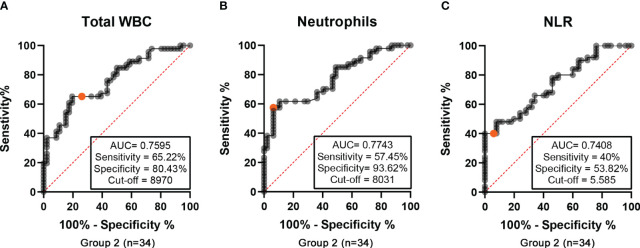
Group 2 (n=34) - Receiver operating characteristic (ROC) curves for longitudinal analysis of BL/LL patients. **(A)** ROC curves comparing total white blood cell (WBC) counts, **(B)** absolute number of neutrophils, and **(C)** neutrophil-to-lymphocyte ratio (NLR) in patients at leprosy diagnosis and at the onset of ENL.

The ROC curves were plotted and the discriminative power levels of the total WBC, neutrophil count, and the NLR were demonstrated (AUC values, 0.7595, 0.7743, and 0.7408, respectively, with a p<0.0001 for all three), as shown in **Figure 5**. The cutoff point was 8970 cells/mm^3^ for the total WBCs (sensitivity 65.22%, specificity 80.43%), 8031 cells/mm3 for neutrophils (sensitivity 57.45%, specificity 93.62%), and 5.585 for the NLR (sensitivity 40%, specificity 53.82%); confirming that ENL is characterized by neutrophilic leukocytosis.

### 3.4 Association between the neutrophilic leukocytosis and severity of ENL

Regardless the ROC curve results for the subgroups 1a and 1b of group 1, as the neutrophil count was significantly higher in the BL/LL ENL subgroup, analysis of the hematology results available for the reactions of patients included in group 3 (ENL group, n=63) was performed, since the ENL reaction was active in this group. A total of 86 ENL reactions with hematological results were analyzed from the 63 patients of this group. The ENL group (group 3, n=63) was further stratified into two categories according to an ENL Severity Scale score based on the EESS (25): a group that presented mild ENL and a group that presented moderate/severe ENL.

**Figure 6:**
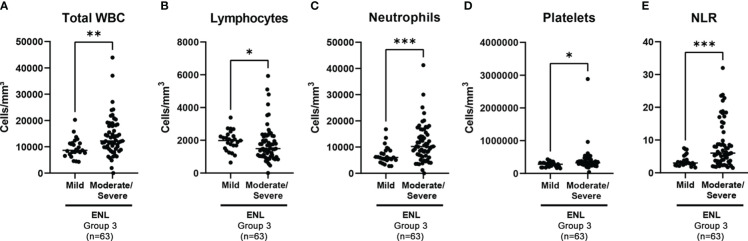
Group 3 (n=63) - Blood cell counts in ENL patients classified as mild and moderate/severe. **(A)** Total white blood cell (WBC), **(B)** lymphocyte, **(C)** neutrophil, and **(D)** platelet counts, and **(E)** neutrophil-to-lymphocyte ratio (NLR) values in mild and moderate/severe ENL. Dots represent individual patients and lines indicate the medians. Statistical analysis was performed using a Mann–Whitney test (*p<0.05; **p<0.01; ***p<0.001).

**Figure 7:**
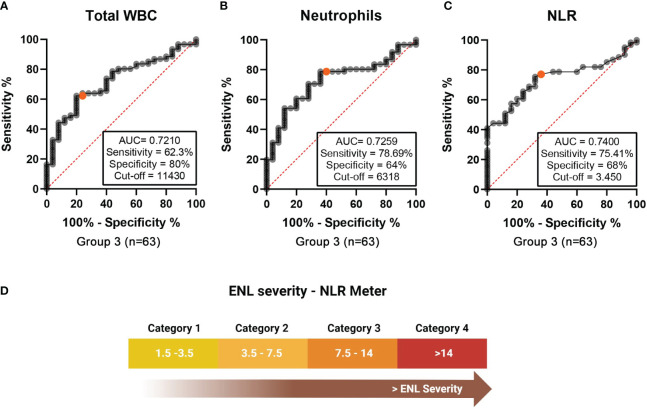
Group 3 (n=63) - Receiver operating characteristic (ROC) curves for blood cells counts in ENL patients classified as mild ENL and moderate/severe ENL. **(A)** ROC curves comparing **(A)** total white blood cell (WBC) counts, **(B)** neutrophil counts, and **(C)** neutrophil-to-lymphocyte ratio (NLR) values in mild vs moderate/severe ENL. **(D)** The ENL meter reflects the NLR values and the severity of the ENL episode. Category 1: patients with NLR values < 3.5. Category 2: NLR values between 3.5 to 7.5. Category 3: NLR ranging from 7.5 to 14. Category 4: NLR > 14.

A comparison was performed between these two stratification categories to verify whether ENL severity had any correlation with the hematological parameters (**Table 6**). A statistical difference was observed between the neutrophil count (6066 cells/mm^3^ for the mild ENL category vs 10243 cells/mm^3^ for the moderate/severe ENL category, p=0.0009); however, no difference was observed in the eosinophil values between the subgroups. Significant differences were also observed between the groups for the WBC, lymphocyte, and platelet counts, and the NLRs (**Table 6** and **Figure 6**). Similarly, ROC curves were plotted and the discriminative power levels of the total WBC count, neutrophil count, and the NLR were considered acceptable (AUC values, 0.7210, 0.7259, and 0.7400; p values, 0.0014, 0.0011, and 0.0005, respectively) (**Figure 7**).

**Table 6:** Group 3 (n=63) - Hematological parameters observed in mild ENL and moderate/severe ENL.

Parameter	Mild ENL	Moderate/severe ENL	p value
RBC count (cells³)b	4500000 (325000–5760000)	4295000 (425000–5490000)	0.1742
Hemoglobin (g/dL)b	12.80 (8.80–15.60)	12.40 (7.20–14.90)	0.1802
Hematocrit (%)b	39.80 (30.40–43.60)	37.40 (23.50–46.00)	0.0516
MCV (µm³)a	86.30 ± 6.067	85.46 ± 6.947	0.5973
MCH (pg)b	28.50 (23.60–32.90)	28.50 (16.90–82.20)	0.7502
MCHC (g/dL)b	32.60 (28.90–37.10)	33.00 (27.50–36.60)	0.2427
WBC count (cells/mm³)b	8630 (4310–20250)	12220 (14.20–43900)	0.0011
Basophils (cells/mm³)b	0.000 (0.000–0.000)	0.000 (0.000–0.000)	>0.9999
Eosinophils (cells/mm³)b	80.10 (0.000–452.4)	0.000 (0.000–2486)	0.2191
Neutrophils (cells/mm³)b	6066 (2758–16808)	10243 (12.10–41266)	0.0009
Lymphocytes (cells/mm³)b	1985 (641.2–3393)	1488 (1.400–5928)	0.0497
Monocytes (cells/mm³)b	537.0 (137.4–1421)	637.6 (0.600–2084)	0.4324
Platelets (cells/mm³)b	280000 (155000–431000)	325000 (43000–2880000)	0.0108
NLR b	3.000 (1.600–7.500)	6.000 (1.500–32.00)	0.0004

ENL, erythema nodosum leprosum; RBC, red blood cell; MCV, mean corpuscular volume; MCH, mean corpuscular hemoglobin; MCHC, mean corpuscular hemoglobin concentration; WBC, white blood cell; NLR, neutrophil-to-lymphocyte ratio; a, Parametric distribution, mean (± standard deviation); b, Non-parametric distribution; median (minimum–maximum). Non-paramertic variables were compared by Mann-Whitnney test. Parametric variables were compared by t-student test.

The cutoff value was selected using the maximum Youden’s index (calculated using the following formula: sensitivity + specificity-1). The cutoff point was 11430 cells/mm3 for the total WBCs (sensitivity 62.3%, specificity 80%), 6318 cells/mm3 for neutrophils (sensitivity 78.69%, specificity 64%), and 3.450 for the NLR (sensitivity 75.41%, specificity 68%); thus, demonstrating a correlation between ENL severity and peripheral blood neutrophil levels.

Next, the NLR values obtained from ENL patients were categorized. Patients with NLR values < 3.5 were classified as Category 1, NLR values between 3.5 to 7.5 were classified as Category 2, NLR ranging from 7.5 to 14 as Category 3, and NLR > 14 as Category 4 (**Figure 7D**).

### 3.5 The relationship between the NLR and the neutrophilic infiltrate in ENL skin lesions

**Figure 8:**
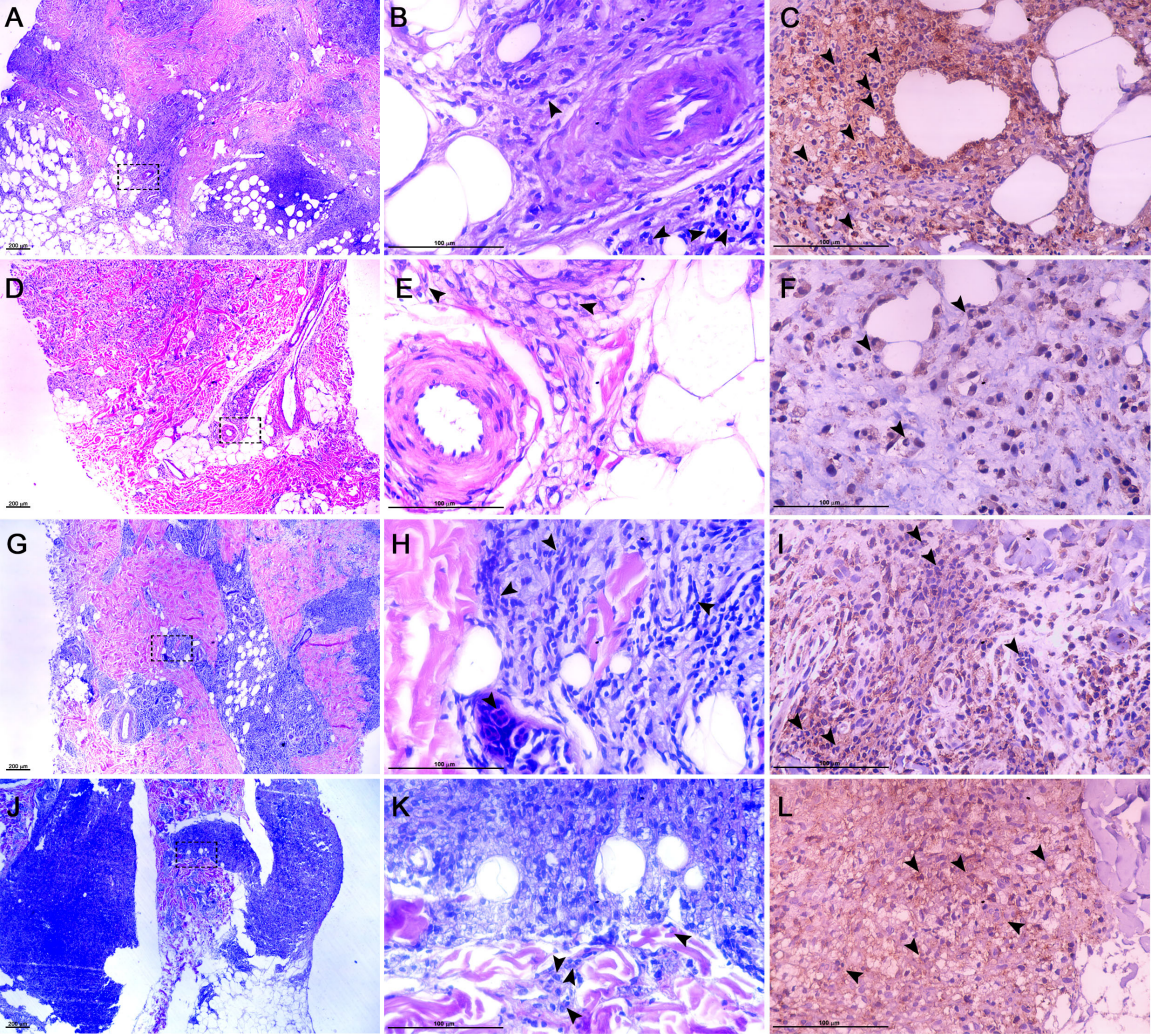
Histology of ENL skin lesions and neutrophils dermal infiltration. HE staining of ENL skin lesions **(A, B, D, E, G, H, J, K)**. High-power representative photomicrographs of the delineated areas indicated by dashed lines in the left column imagens **(B, E, H, K)**, black arrowheads indicate neutrophils in reticular dermal tissue areas. Distribution of neutrophilic dermal infiltrate confirmed by pentraxin-3 expression (*black arrowheads* - **C, F, I, L**). Images represented the categorization based on NLR values and the distribution of neutrophilic dermal infiltration. Images are representative of 8 ENL patients. All images were obtained via Nikon Eclipse microscope with Opticam Microscopia OPTHD software. Scale bar: 200 μm **(A, D, G, J)** and 100 μm **(B, C, E, F, H, I, K, L)**.

To ascertain the correlation between NLR and the presence of neutrophils in ENL tissue sections, 43 ENL samples were analyzed. Tissue sections were assessed based on the intensity of neutrophilic dermal infiltration, categorized as mild, moderate, and intense. Representative images from four tissue sections of different ENL patients are presented (**Figure 8**).

Varying numbers of eosinophils have also been described in ENL skin lesions (17). To confirm the presence of neutrophils in skin lesions of ENL patients, immunohistochemistry analysis was performed using Pentraxin-3 (PTX3), a marker of neutrophils (26). The immunohistochemistry results confirmed that inflammatory infiltrated observed in ENL skin lesions is composed by neutrophils, since eosinophils do not contain preformed PTX3 (**Figures 8C, F, I, L**).

Each of the 43 ENL skin samples had a corresponding hematological result. Consequently, every reaction was linked with the NLR value. ENL patients were categorized based on their NLR values to determine if there was a relationship between NLR and the presence of neutrophilic dermal infiltration.

**Table 7** presents the frequency of patients in each category and the corresponding intensity of neutrophilic infiltration. Results show that patients with ENL classified as Category 4 (NLR > 14) had more frequency of skin lesions with a more intense infiltration of neutrophils (**Figures 8D–F**).

**Table 7:** Categorization of erythema nodosum leprosum lesion biopsies according to NLR levels.

Category	NLR	Mild (%)*	Moderate (%)*	Intense (%)*
1	1.5–3.5	33.33	53.33	13.33
2	3.5–7.5	30.77	38.46	30.77
3	7.5–14	62.50	25.00	12.50
4	>14	14.29	42.86	42.86

NLR, neutrophil-to-lymphocyte ratio; *Percentages correspond to the intensity of the neutrophilic infiltrate.

## 4 Discussion

The Neutrophil-to-lymphocyte ratio (NLR) is a measure that shows how the innate (neutrophils) and adaptive (lymphocytes) immune systems interact in the body’s physiological and pathophysiological responses. NLR is a widely used marker of immune response and is considered reliable and easily accessible across almost many medical conditions (23). In the present study, we characterized the demographic, clinical, and hematological profiles of a retrospective cohort of patients affected by BL or LL leprosy and demonstrated that neutrophilic leukocytosis is a characteristic component of ENL. A longitudinal analysis showed that the neutrophil count and the NLRs were higher during ENL than before the start of MDT. Interestingly, an analysis of blood counts before the start of MDT showed that those who developed an episode of ENL already had higher levels of circulating neutrophils than those that did not. This correlation was also demonstrated by the direct relationship between the severity of ENL and the levels of neutrophils observed in the blood counts of patients during the reactional episode.

Our results for the longitudinal assessment demonstrated a NLR of 6.564 in patients with ENL versus 2.839 in patients with BL/LL leprosy. These values are in line with those of Gomes et al. (2020), who evaluated the NLR as a diagnostic biomarker of the different reaction states in patients with leprosy and concluded that the highest NLR was observed in ENL (19). Tanojo et al.(2022) reported that the NLR had an 82.95% accuracy in diagnosing the occurrence of ENL in a retrospective study of 182 patients with MB leprosy (27).

In our cohort, most of the patients with leprosy presented multiple episodes of ENL. A large proportion of cases were categorized as chronic ENL, and 62% of cases occurred after release from MDT, 1.6 years after the beginning of the treatment on average. Differently, Negera et al. (2017) reported a lower proportion of chronic ENL (39%) in their study with 77 patients in a hospital at Ethiopia (16). Our results are similar to those of a systematic review performed by Voorend et al. (2013), who demonstrated that multiple episodes of ENL were found in 39%–77.3% of patients with MB leprosy recruited, with patients experiencing an average of 2.6 episodes. On the other hand, the authors indicated that the incidence of ENL during MDT was at least twice as high as at the time of the initial leprosy diagnosis (28), which was not the case in the present cohort. The higher prevalence of ENL in our facility is probably because it is a reference center for leprosy in Rio de Janeiro, which receives more complex and severe cases.

In this study, we confirmed that the BI is a contributing risk factor to the development of ENL, as 95% of those who presented an ENL episode had a BI of >3 upon leprosy diagnosis. A previous study conducted in the same facility in 1998 demonstrated similar results (9). Balagon et al. (2011) also showed that a high initial BI is the key risk factor for ENL (29). Indeed, a systematic review conducted by Voorend and Post (2013) concluded that the main risk factors for developing ENL are a high bacteriological index and a borderline lepromatous/lepromatous (BL/LL) classification in the Ridley-Jopling spectrum (28).

Neutrophils are the most prevalent type of WBC, safeguarding the body through defense mechanisms, such as phagocytosis, the release of antimicrobial peptides, and neutrophil extracellular traps (11). A perivascular influx of neutrophils throughout the dermis and subcutis has been observed in ENL lesion biopsies, especially within the initial 72h of onset (11, 28). Circulating neutrophils of patients before MDT contain *M. leprae*, even in the absence of systemic inflammation and presenting leprosy reactions (30).

The significance of neutrophils in leprosy has often been overlooked, with numerous studies focusing on the macrophages and Schwann cells impacted by *M. leprae* (15). This oversight may be attributed to the fact that neutrophils are not the primary targets of the mycobacteria, unlike the aforementioned cells (31). However, recent studies on neutrophils have indicated an active role for these cells in ENL, as opposed to a passive one. These findings offer fresh insights into the involvement of neutrophils in the disease (15). Components of neutrophil granules, such as myeloperoxidase (MPO) and matrix metalloproteinase (MMP)-9, have also been observed in blood during ENL episodes (30). Lee et al. identified a genetic signature associated with neutrophil recruitment in ENL lesions, emphasizing P-selectin, E-selectin, and their ligands (32). Our research group also showed increased expression of genes and proteins associated with neutrophils in patients with ENL, emphasizing CD64 (a neutrophil marker of activation) and PTX-3 (33, 34). The release of neutrophil extracellular traps (NETs) in circulation and cutaneous lesions of ENL patients suggests that neutrophil activation may contribute to the systemic inflammation observed in ENL (35), which could be the source of DNA that stimulates mononuclear cells described by Dias et al., 2014 (36). Furthermore, *M. leprae* also induces neutrophil degranulation (31) and cytokine release (37) *in vitro*. All neutrophil components, including DNA, protein of granules, and cytokines, may contribute to the storm of cytokines observed in ENL patients. Recently, data obtained by whole blood transcriptomic analyses of ENL patients demonstrated enrichment of neutrophil activation and degranulation-related genes, with the neutrophil activation marker CD177 being the most enriched gene of ENL episode (38).

MDT, which causes bacterial fragmentation, is hypothesized to trigger the formation of antigen-antibody complexes in ENL (11). Wemambu and Turk (1969) described granular deposits of immunoglobulins and complement in ENL skin lesions (39). Recently, Negera et al. (2018) demonstrated elevated levels of C1q, the first protein of the complement classical pathway, in skin lesions of untreated ENL patients when compared to non-reactional LL patients. Interestingly, circulating C1q in the peripheral blood of untreated ENL patients was significantly decreased compared to LL patient controls (40). Deposits of M. leprae antigens have also been described in the dermis and in areas where neutrophil infiltration is found. Two of the MDT components, dapsone and clofazimine, have been related to changes in the function of neutrophils and lymphocytes, as dapsone stimulates neutrophil migration and inhibits lymphocyte transformation (41–43).

To evaluate the predictive biomarker value, we compared the hematological results from treatment-naive BL/LL patients that were NR or had ENL and found a statistical difference in the eosinophil median counts. Inyang et al. (2022) reported that patients with a higher absolute eosinophil count presented 9.11 odds of developing leprosy reactions than those with normal or low values (44). However, in the present study, the eosinophil values were lower in the ENL subgroup than in the NR subgroup. This comparison also revealed that, even before starting MDT, the neutrophil values from patients that developed ENL after beginning MDT, were higher than those who did not experience any reaction, suggesting that this could predict pre-existing condition for ENL development.

Most of the ENL episodes that we were able to categorize according to the Severity were moderate/severe (78%). Data from this stratification showed a significant elevation in the total WBC count with significant neutrophilia and lymphopenia in patients with moderate/severe ENL, which was also observed by Vaishnani et al. (2023) and Tanojo et al. (2022) (27, 45). Our results confirmed the correlation between the increase in neutrophil counts and ENL severity: the median neutrophil count for mild ENL was significantly lower than for moderate/severe ENL, and the median NLR was higher in moderate/severe ENL than in mild ENL, with a diagnostic cutoff value of 3.4 for severity assessment. The study performed by Vaishnani et al. (2023) had already demonstrated this difference in WBC, neutrophil, lymphocyte, and NLR values (45); however, these values cannot be compared because neither Vaishnani nor our study adopted the EESS.

The elevation in platelet counts observed in the moderate/severe ENL subgroup may be associated with the impact of cytokines that are produced during an ENL episode on the bone marrow, leading to increased synthesis and release into the peripheral blood (46). A study with 18 patients also reported a significantly higher mean platelet count in cases of moderate to severe ENL compared to that in mild cases (47). Other authors have described this pattern (19, 27, 47), but did not correlate this with the disease severity.

The longitudinal assessment performed in the group of patients with hematological results before MDT and at ENL diagnosis demonstrated neutrophilic leukocytosis in ENL. This was confirmed by elevated neutrophil values (BL/LL: 4896 cells/mm3 vs. ENL: 8408 cells/mm3, p<0.0001) and a statistical difference in the NLR between the two time points (BL/LL: 2.570 vs. ENL: 3.900, p<0.0001). Gomes et al. (19) likewise reported this difference, however, with a cutoff of 2.95 to diagnose ENL compared with 5.59 in the present study.

A major histological hallmark of ENL skin lesions is the prominent neutrophilic infiltrate, mainly within the deep layers of the dermis and subcutaneous tissue superimposed on LL lesion.

The relationship between NLR and neutrophilic infiltrate in skin lesions was further investigated. Our analysis observed that the higher the NLR, the more intense the neutrophilic infiltrate in the ENL lesion. This may explain why, in our results, some ENL biopsies do not show this histological marker and why some blood samples from patients with ENL present low NLR values. Although neutrophilia is associated with ENL, the primary trigger for neutrophil expansion in peripheral blood is unknown, and it is impossible to monitor how and when this movement of neutrophils from the bone marrow to the blood and skin occurs during the reaction. The presence of *M. leprae* in the bone marrow could increase the granulopoiesis, as well as bacillary antigenic load and increased immunoglobulins level owing to predominant humoral response results in immune complex formation suggest systemic inflammatory response and results of the proliferative effect of inflammatory mediators on bone marrow cells (37).

The sample size of the present study was limited (252 patients from a single center), and the clinical, histopathological, and hematological data were retrospectively analyzed. Despite this, the findings appear to align with those of other studies. Given that blood counts are routine in clinical practice, the reproducibility of these results should be performed in other centers, particularly in prospective studies. These findings could aid in improving ENL management, thereby averting potential complications and reducing the patients’ health-related quality of life.

The histopathological results and their association with NLR, a novel aspect of our research, provide further insights into the relationship between circulating neutrophil and neutrophilic infiltrate in skin lesions of leprosy patients with ENL.

## Data availability statement

The original contributions presented in the study are included in the article/supplementary material. Further inquiries can be directed to the corresponding author.

## Ethics statement

The studies involving humans were approved by Fiocruz/IOC Human research ethics Review Board. The studies were conducted in accordance with the local legislation and institutional requirements. The ethics committee/institutional review board waived the requirement of written informed consent for participation from the participants or the participants’ legal guardians/next of kin. As this was a retrospective study, the research relies on data that has already been collected from a database and medical records. After leprosy treatment completion, almost all participants ended their follow-up at ASA outpatient clinic, and in many cases, (1) patients no longer regularly attend the clinic service, or, (2) the address and telephone numbers are no longer the same and there are no other forms of contact. Notwithstanding the researchers were not actively involving participants in any new data collection process. The data used in the research was anonymized to protect the privacy and confidentiality of individuals.

## Author contributions

MF: Writing – review & editing, Writing – original draft, Project administration, Methodology, Investigation, Formal analysis, Conceptualization. MA: Writing – review & editing, Methodology. AMS: Writing – review & editing, Investigation. DF: Writing – review & editing, Investigation. DP: Writing – review & editing, Investigation. DJ: Writing – review & editing, Investigation. TL: Writing – review & editing. IF: Writing – review & editing, Methodology. HF: Formal analysis, Methodology, Writing – review & editing. TP: Formal analysis, Methodology, Writing – review & editing. MG: Writing – review & editing, Investigation. AMM: Writing – review & editing, Investigation. XI: Writing – review & editing, Methodology. VS: Writing – review & editing, Writing – original draft, Supervision, Resources, Methodology, Funding acquisition, Conceptualization.
